# Selinexor in acute myeloid leukemia: therapeutic applications and current challenges

**DOI:** 10.3389/fphar.2025.1602911

**Published:** 2025-05-20

**Authors:** Ziyu Qie, Li Ma, Jie Tan, Xiaochun Peng

**Affiliations:** ^1^ Department of Hematology, The First Affiliated Hospital of Yangtze University, Jingzhou, China; ^2^ Department of Gastroenterology, Jianli People’s Hospital, Jingzhou, China; ^3^ Laboratory of Oncology, Center for Molecular Medicine, School of Basic Medicine, Health Science Center, Yangtze University, Jingzhou, China

**Keywords:** selinexor, acute myeloid leukemia, XPO1 inhibitor, treatment strategy, drug resistance

## Abstract

This article aims to review the current application status and research advancements of selinexor in the treatment of acute myeloid leukemia (AML). Selinexor, as the first oral selective inhibitor of nuclear export protein Exportin-1 (XPO1), inhibits the abnormal nuclear export of tumor suppressor proteins by blocking XPO1, thereby restoring their activity and exerting antitumor effects. Clinical studies have shown that selinexor monotherapy or combination therapy has demonstrated good anti-leukemia effects in AML, especially in patients with relapsed/refractory AML. In addition, the combination of selinexor with other drugs, such as demethylating agents and FLT3 inhibitors, has shown synergistic antitumor effects. Although selinexor has shown potential, its resistance and adverse reactions still need further research and control. Future research directions include exploring the best medication schemes, clarifying the appropriate population, and developing new combination treatment plans to improve treatment effects and overcome drug resistance issues.

## 1 Introduction

AML is a clonal stem cell cancer characterized by the proliferation and maturation arrest of immature myeloid precursor cells, leading to bone marrow failure (Venugopal and Sekeres, 2024). The clinical manifestations of AML are diverse, encompassing fatigue, dizziness, palpitations, dyspnea, infections or fever. Additionally, patients may present with hepatosplenomegaly, mucosal bleeding, skin or testicular masses, skin infiltration, gum enlargement, bone pain, cutaneous chloroma, and abdominal pain. The annual incidence rate of AML is 4.1 per 100,000 population. In terms of gender, the incidence rate in males (5.0 per 100,000) is higher than in females (3.4 per 100,000). The median age at diagnosis is 69 years. In people aged 70 and above, the annual incidence rate increases to 17.3 per 100,000 (Venugopal and Sekeres, 2024).

The current treatment methods for AML mainly include standard chemotherapy, targeted therapy, supportive treatment, and hematopoietic stem cell transplantation (Fang et al., 2024). In standard chemotherapy, induction chemotherapy is first performed, using cytarabine and anthracycline drugs (such as daunorubicin or idarubicin) for treatment, followed by other high-intensity or low-intensity chemotherapy regimens for consolidation of effects (Pollyea et al., 2023). Targeted therapies can be used for AML with high heterogeneity in cellular genomic characteristics (Network et al., 2013), such as FLT3 inhibitors (such as midostaurin (Stone et al., 2017), quizartinib (Erba et al., 2023), sorafenib (Xuan et al., 2020), and gilteritinib (Perl et al., 2019; Pratz et al., 2020) for patients with FLT3-mutated AML, IDH1 inhibitors (ivosidenib (DiNardo et al., 2018) and olutasidenib (De Botton et al., 2023) for patients with IDH1-mutated AML, and IDH2 inhibitors (such as enasidenib (Stein et al., 2017) for patients with IDH2-mutated AML. For elderly patients or those with comorbidities who cannot tolerate intensive chemotherapy, there are hypomethylating agents [such as azacitidine (Wei et al., 2020) and decitabine] or combined Bcl-2 inhibitor regimens available (DiNardo et al., 2020). Emerging alternative therapeutic strategies are also being explored. For instance, recent studies highlight the potential of RNA nanotechnology in enhancing drug targeting and reducing systemic toxicity, which may complement existing AML therapies (Fang et al., 2024). Subsequent supportive treatments (transfusions, anti-infective treatments, growth factor support, etc.) and hematopoietic stem cell transplantation (HCT) are also effective means to improve cure rates (Pollyea et al., 2023).

In terms of prognosis for AML patients, the 5-year survival rate for AML is 31.7% (De Botton et al., 2022). For relapsed/refractory AML (R/R AML), the long-term survival rate treated with traditional chemotherapy regimens is only 30%–40%, with a cure rate of less than 10% (Bose et al., 2017). The overall survival (OS) rate for childhood acute myeloid leukemia (AML) can reach 60%, and the 5-year event-free survival (EFS) rate is over 50% (Sakamoto et al., 2014). Data analysis from the MD Anderson Center suggests that for elderly (aged ≥65) AML, the remission rate with intensive chemotherapy is 40%–50%, with an early mortality rate of 26%–36% within 1–2 months, a median survival of only 4–6 months, and a 1-year overall survival (OS) rate below 30%, which is significantly lower compared to young patients with a remission rate of 70%–80% and a long-term survival rate of 40%–50% (Kantarjian et al., 2021). Therefore, despite the diversity of current treatment options, AML still has characteristics of low effective rates for relapsed/refractory AML and poor prognosis for elderly patients, with unmet treatment needs. New treatment strategies are required to improve patient cure rates and quality of life.

**FIGURE 1:**
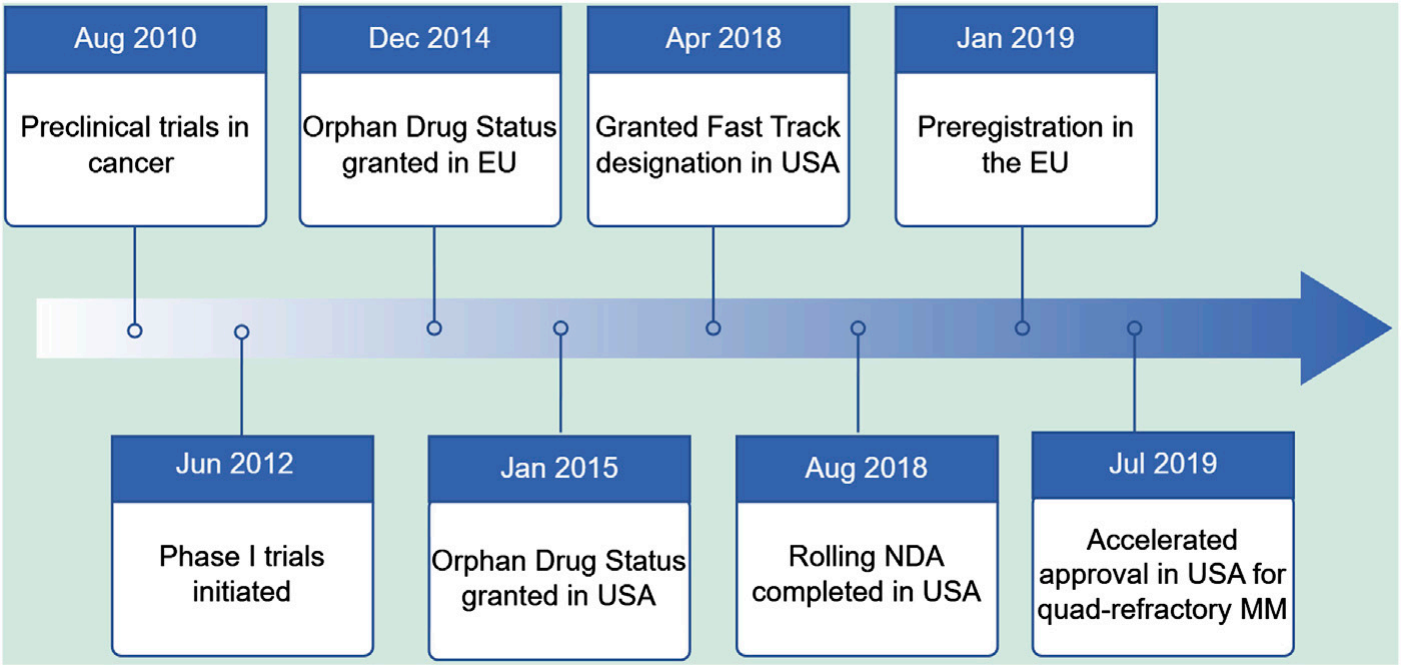
Key milestones in the development of Selinexor.

In this context, researchers have begun to focus on the role of nucleocytoplasmic transport proteins in the development and progression of tumors, especially the nuclear XPO1. XPO1 is the most critical and widely studied nucleocytoplasmic transport protein in cells. Previous studies confirmed that XPO1 is overexpressed in various hematological tumors and solid tumors, and is associated with disease progression, drug resistance, and prognosis (Lin and Wang, 2022), suggesting it as a potential target for cancer treatment. Since the discovery of the first XPO1 inhibitor in 1997, research on the XPO1 target has undergone decades of exploration. The development of nuclear export protein inhibitors has progressed rapidly, initially with preliminary attempts of drugs such as leptomycin B, anguinomvcins, and ratjadones, but due to irreversible binding and low activity leading to significant adverse reactions, they did not enter the clinic (Gui et al., 2024; Lapalombella et al., 2012; Newlands et al., 1996). Subsequent computer-aided drug design led to a series of highly selective nuclear export protein inhibitors (Selective Inhibitors of Nuclear Export, SINE), such as KPT-330 (selinexor), KPT-335 (verdinexor), and KPT-8602 (eltanexor), which bind slowly and reversibly to the leucine-rich nuclear export signal (NES) binding site of XPO1, have good pharmacokinetic parameters, high oral bioavailability, and mild adverse reactions (Parikh et al., 2014; Mahipal and Malafa, 2016). Among them, selinexor has gradually become the focus of treating hematological malignancies after a series of development processes (Figure 1). Today, the U.S. Food and Drug Administration (FDA) has approved selinexor for the treatment of relapsed/refractory multiple myeloma (MM) and diffuse large B-cell lymphoma (DLBCL). In China, the National Medical Products Administration (NMPA) approved selinexor in combination with dexamethasone for the treatment of relapsed/refractory MM on 14 December 2021.

XPO1 inhibitors trap tumor suppressor proteins within the nucleus, restore apoptotic signaling, and exert antitumor effects. They also work synergistically with multiple drugs to enhance antitumor activity. The exploration of XPO1 inhibitors in the targeted treatment of AML has become a research hotspot in recent years. Compared with other emerging AML therapies, Selinexor has also shown unique advantages. For example, compared with FLT3 inhibitors (such as Midostaurin, Quizartinib, etc.), Selinexor can overcome the problem of resistance to FLT3 inhibitors (Zhang et al., 2018). By inhibiting the function of XPO1, it restores the activity of tumor suppressor proteins, thereby enhancing the anti-leukemia effect (Zhang et al., 2018). In addition, when used in combination with BCL-2 inhibitors (such as Venetoclax), Selinexor can further enhance apoptosis and overcome the limitations of monotherapy (Long et al., 2023). However, these combination therapies still need to be further verified for their safety and efficacy in clinical trials.

Despite the progress made in the treatment of AML, many challenges remain unresolved. For patients with relapsed/refractory AML, the long-term survival rate of traditional chemotherapy regimens is still very low, and the prognosis of elderly patients is poor (Bose et al., 2017). In addition, current therapeutic approaches still have limitations in terms of efficacy and safety. For elderly patients or those with comorbidities who cannot tolerate intensive chemotherapy, there are limited treatment options (Kantarjian et al., 2021). Therefore, there is an urgent need for new therapeutic strategies to improve patients’ cure rates and quality of life. This article reviews the research progress of selinexor in the field of AML and analyzes potential therapeutic combinations and research directions that can be further explored.

## 2 Selinexor’s mechanism of action and pharmacological properties

### 2.1 Selinexor’s mechanism of action

XPO1, also known as chromosome region maintenance 1 (CRM1), recognizes and binds to proteins containing nuclear export signals (NES) and, under the direction of energy and directionality provided by Ran GTPase proteins, promotes the export of these proteins from the nucleus to the cytoplasm through the nuclear pore complex (NPC) (Nachmias and Schimmer, 2020). XPO1 primarily exports tumor suppressor proteins (TSPs), including p53, p21, and FOXO3A. The mislocalization of these proteins can disrupt their antitumor functions (Nachmias and Schimmer, 2020). The abnormal nuclear export of the above proteins leads to the inactivation of protein function, increased drug resistance, and abnormal proliferation and differentiation of tumor cells (Hutten and Kehlenbach, 2007; Gravina et al., 2014). Kojima et al. (Kojima et al., 2013) detected the expression level of XPO1 protein in 511 *de novo* AML patients and variable analysis showed that high expression of XPO1 is an independent predictor of overall survival (OS), which is related to high-risk cytogenetics and shorter survival.

**FIGURE 2:**
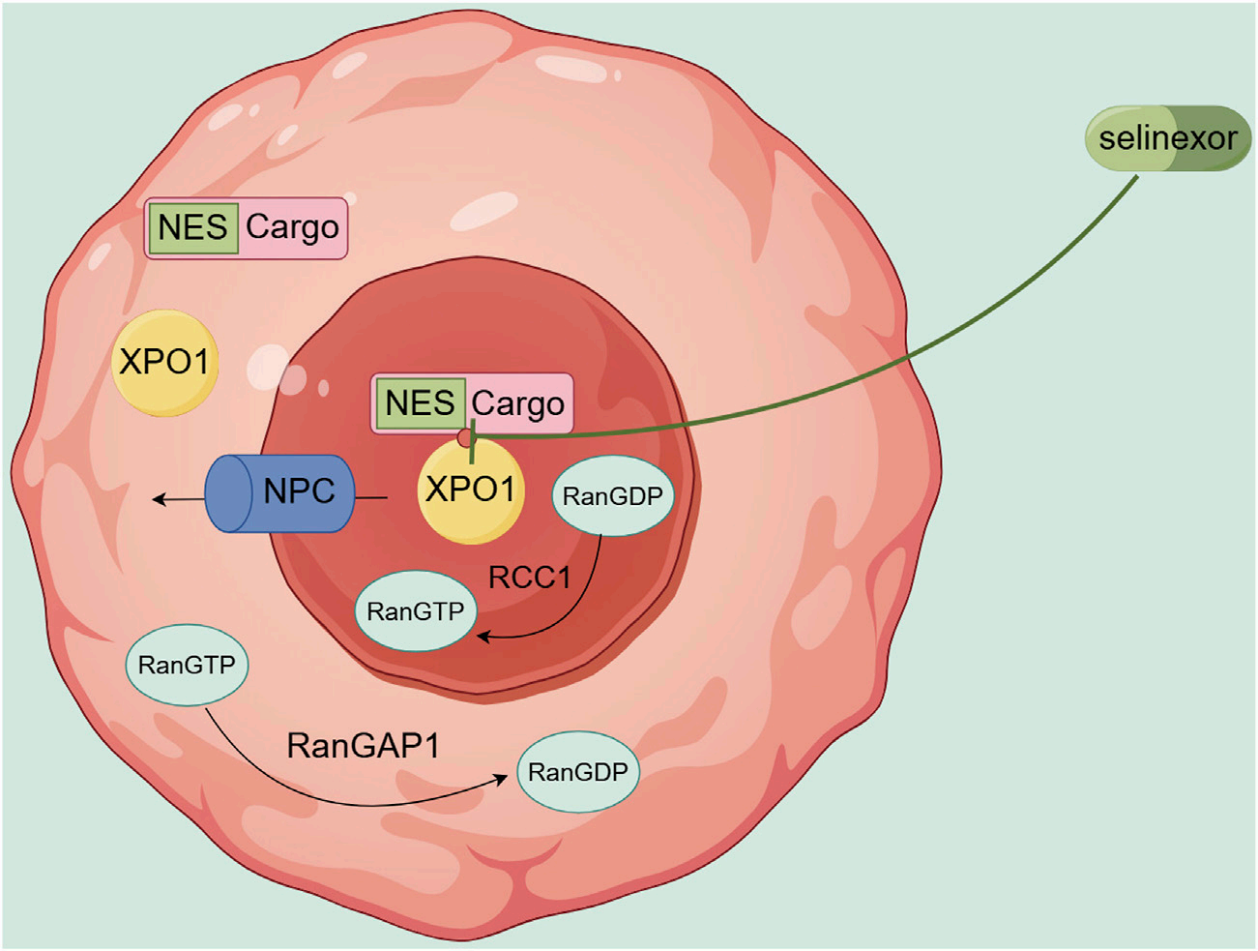
Transport mechanism of XPO1 and site of action of Selinexor.

However, selinexor (Selinexor), a selective inhibitor of nuclear export protein XPO1 and a highly selective, reversible binding small molecule oral nuclear export protein inhibitor compound (SINE), can covalently bind to the cysteine 528 site in the leucine-rich nuclear export signal (NES) binding region of XPO1, slowly and reversibly inhibiting the nuclear export function of XPO1(Figure 2), restoring the activity of TSP, and playing a role in promoting apoptosis and anti-tumor activity, while allowing a certain degree of nuclear export to ensure the survival and function of normal cells (Grayton et al., 2017; Etchin et al., 2016). Selinexor exhibits remarkable cytotoxicity against AML cells, while exerting no apparent effects on normal hematopoietic stem cells (Etchin et al., 2013) and progenitor cells (Etchin et al., 2015). Experimental studies have shown that in AML, XPO1 inhibitors can act on the following key TSP.

(1) TP53: TP53 is one of the most common mutated genes in hematological tumors, regulated by MDM2 (an E3 ubiquitin ligase) (Kojima et al., 2013). TP53 mutation is related to drug resistance and poor prognosis in AML treatment. XPO1 inhibitors can induce the nuclear retention of p53, synergize with MDM2 inhibitors to block the ubiquitination and degradation of p53, and maximize the activation of p53-mediated apoptosis (Kojima et al., 2006; Kojima et al., 2005).
(2) NPM1: More than 30% of AML patients have NPM1 mutations, which often coexist with other AML pathogenic gene mutations. NPM1 mutation leads to the replacement of the nuclear localization signal (NLS) with NES, thereby transporting it out of the nucleus and upregulating the expression of HOX/Meis1 genes, maintaining the leukemia state (Spencer et al., 2015; Alcalay et al., 2005). XPO1 inhibitors induce their cells, reduce the expression of HOX/Meis1, and promote the differentiation of AML cells (Kojima et al., 2013; Brunetti et al., 2018).
(3) BCL2, MCL1: Basic research has shown that venetoclax, a BCL2 inhibitor, selectively acts on BCL2, and MCL1 compensatory upregulation leads to its resistance, which is particularly evident in relapsed/refractory AML (R/R AML) patients. XPO1 inhibitors inhibit the nuclear export translation process of BCL2 and MCL1 mRNA mediated by eIF4E (eukaryotic initiation factor 4E), simultaneously downregulate the expression of BCL2 and MCL1 proteins, and induce apoptosis in AML cells (Fischer et al., 2020; Luedtke et al., 2018).
(4) FLT3: FLT3-ITD mutation is related to poor prognosis, and although FLT3 inhibitors have certain efficacy, FLT3 receptor gene mutations can reduce the binding force of FLT3 inhibitors or cause compensatory activation of downstream signaling pathways, leading to drug resistance. Studies have shown that AML cells with FLT3 mutations are more sensitive to XPO1 inhibitors than wild-type FLT3, and dual targeting of XPO1 and FLT3 can produce synergistic effects, significantly extending the survival of AML mice (Zhang et al., 2018).

In addition, studies have shown that XPO1 inhibitors can downregulate the expression levels of DNA repair genes (Rad51 and Chk1) dependent on c-myc gene, inhibit homologous recombination repair of tumor cells, and promote the nuclear localization of Topo IIa, restoring the drug sensitivity of Topo IIa inhibitors (Ranganathan et al., 2016). Ranganathan et al. (Ranganathan et al., 2015) also found that sequential treatment of AML cells with decitabine followed by XPO1 inhibitors can upregulate the expression of CDKN1A and FOXO3A, enhance anti-leukemia activity, and significantly extend the survival of AML mice compared to monotherapy with selinexor. The above preclinical studies provide theoretical support for the clinical research exploration of XPO1-targeted therapy in the AML field. As the only approved selective XPO1 inhibitor, selinexor has undergone extensive early clinical research in the AML field.

### 2.2 Pharmacological properties of selinexor

Selinexor is rapidly absorbed after oral administration, with an average time to reach maximum concentration of 2–4 h (Gounder et al., 2016; Razak et al., 2016; Alexander et al., 2016). Studies have shown that the peak induction of XPO1 mRNA occurs 4–8 h after the initial administration of selinexor, and its elevated levels persist for 24–48 h (Garzon et al., 2017). This means that selinexor can slowly and continuously inhibit XPO1 for up to 48 h after achieving target occupancy. The possible reason is the effect achieved by the overlapping half-life of multiple selinexor doses. Selinexor is mainly metabolized by the liver and eliminated by the biliary system, with an elimination half-life of 6–8 h. Mild liver damage does not have clinical significance on the pharmacokinetics of selinexor. The population pharmacokinetic data from phase I to II studies showed no significant changes in the pharmacokinetics of selinexor in 13 patients with moderate to severe liver damage. The kidney is not the main route of elimination, and no dose adjustment is needed for patients with renal insufficiency. Selinexor is mainly metabolized by cytochrome P450 3A4 (CYP3A4), various UDP-glucuronosyltransferases (UGT), and glutathione S-transferases (GST). Population pharmacokinetic data evaluated the impact of co-medication on selinexor exposure and found that CYP3A4 weak, moderate, and strong inhibitors, CYP3A4 inducers, CYP2D6 inhibitors, CYP2C8 inhibitors, proton pump inhibitors (PPI), H2 receptor blockers, and dexamethasone do not affect the pharmacokinetic data of selinexor (Bader et al., 2021). As of December 2022, in more than 3,200 patients treated with selinexor alone or in combination, there have been few reports of clinically significant drug interactions. Therefore, the co-use of the above drugs does not affect the exposure of selinexor, and no dose adjustment is needed. In addition, preclinical data show that the blood-brain barrier permeability of selinexor in rats and monkeys is 60%–80% (Green et al., 2014).

The above pharmacokinetic characteristics determine the concentration and action time of selinexor in the body, which in turn affects its efficacy. For example, its moderate half-life and clearance rate mean that patients can receive regular treatment without the need for too frequent dose adjustments.

## 3 Clinical studies of selinexor in the treatment of acute myeloid leukemia

### 3.1 Selinexor monotherapy for AML

This section will focus on the efficacy and prognosis of selinexor monotherapy for AML. For detailed research data, refer to Table 1. Preclinical trials showed that selinexor was notably toxic to AML cells in NSG mice, reducing human AML cells in the bone marrow and spleen, especially leukemia-initiating cells (LICs), while minimally affecting normal hematopoietic cells, thus preserving normal hematopoiesis (Etchin et al., 2015). Based on these results, a Phase I trial for relapsed/refractory AML (n = 95) was initiated. In a study of 81 patients, the recommended dose of selinexor for treating relapsed/refractory AML was set at 60 mg twice weekly. This dose showed good efficacy and safety, with most adverse events being mild to moderate. Further analysis indicated that patients with lower initial bone marrow blasts were more likely to respond to selinexor, suggesting it may be more suitable for those with MDS or hypoproliferative AML (Garzon et al., 2017). Another Phase II trial recruited R/R AML patients aged over 60 who were ineligible for intensive chemotherapy or bone marrow transplantation. Compared with the investigator’s choice of treatment, selinexor monotherapy demonstrated a significantly higher complete remission/complete remission with incomplete blood count recovery (CR/CRi) rate. Additionally, the study suggested that the activity levels of five proteins might be correlated with selinexor efficacy, and TP53 mutations were associated with an unfavorable prognosis (Sweet et al., 2021). A Phase I study evaluated the safety and efficacy of selinexor monotherapy in patients who underwent allogeneic hematopoietic stem cell transplantation. The findings suggested that long-term selinexor maintenance therapy could be a safe and feasible option for high-risk AML and MDS patients. However, some patients developed acute graft-versus-host disease (aGVHD), which may not have been directly related to selinexor treatment. Additional research is warranted to further explore these findings (Cooperrider et al., 2020).

**TABLE 1:** Summary of clinical studies of Selinexor (X) monotherapy in patients with acute myeloid leukemia (AML).

Literature	Study phase	Number of cases	Patient characteristics	Study protocol	X Dose	CR/CRi [Cases (%)]	PFS (Months)	OS (Months)	Transplantation rate (%)	Trial registration number
Garzon et al.	Phase Ⅰ	95	R/R AML	X monotherapy with six dose levels (16.8–70 mg/m^2^), 21 days or 28 days as 1 cycle	60mgBIW, 4 weeks per course	7/81 (9%)	1.7	2.7	—	NCT01607892
Sweet et al.	Phase Ⅱ	118 vs. 57	≥60 years R/R AML	X monotherapy vs. physician’s choice	60mgBIW, 4 weeks per course	11.9% vs. 3.5%	—	3.2 vs. 5.6	—	NCT02088541
Cooperrider et al.	Phase Ⅰ	12	AML and MDS post-transplant	X monotherapy (60–80 mg QW) for 12 courses	60mgQW, 4 weeks per course	9/12 (75%)	2-year PFS rate 50%	—	—	NCT02485535

Notes: RP2D: Recommended Phase II, dose; R/R: Relapsed/Refractory; MDS: myelodysplastic syndromes; BIW: twice a week; QW: once a week; CR: complete remission; CRi: Complete Remission with Incomplete Hematologic Recovery; PFS: Progression-Free Survival; EFS: Event-Free Survival; OS: overall survival; -: no data available.

### 3.2 Selinexor combination therapy for AML

This part will focus on the choice and clinical outcomes of selinexor combination therapies with various drugs for AML. For specific data, refer to Table 2. Phase I and II clinical studies have explored combining selinexor with chemotherapy for R/R AML. In a Phase II trial, selinexor with cytarabine and idarubicin treated 42 R/R AML patients, achieving CR/CRi in 20, and offering bone marrow transplant opportunities. Low - dose selinexor with chemotherapy also showed good tolerability (Fiedler et al., 2020). Another Phase I trial combined selinexor with the FLAG-ida regimen in young R/R AML patients. Some fatal adverse events occurred. But efficacy was shown with a response rate of 42%. As the MTD wasn’t reached and 100 mg/week of selinexor showed acceptable tolerability and better efficacy, the RP2D was set at 100 mg/week (Martínez Sánchez et al., 2021). This article also analyzed the relationship between gene mutation status and treatment response in AML patients, finding no clear correlation but noting a lower CR/CRi rate in patients with higher bone marrow blasts. It also reported that combining selinexor with the CLAG regimen was safe and effective, serving as a bridge to transplantation for R/R AML patients (Abboud et al., 2019).

**TABLE 2:** Summary of clinical studies of Selinexor (X) combination therapy in patients with acute myeloid leukemia (AML).

Literature	Study phase	Number of cases	Patient characteristics	Study protocol	X dose	CR/CRi [Cases (%)]	PFS (Months)	OS (Months)	Transplantation rate (%)	State	Trial registration number	Remarks
Fiedler et al.	Phase Ⅱ	42	R/R AML	X (40 mg/m^2^ and 60 mg BIW) combined with IDA (10 mg/m^2^, days 1, 3, 5) and Ara C (100 mg/m^2^, days 1–7)	60mgBIW, 3 weeks on, 1 week off	20/42 (47.6%)	—	8.2	35.7	Completed	NCT02249091	It is most promising when combined with cytarabine and anthracyclines
Martínez Sánchez et al.	Phase Ⅰ	14	R/R AML	X (60–100 mg BIW) combined with FLAG-IDA	100mgQW, 3 weeks on, 1 week off	5/12 (42%) RP2D group 66.7%	mEFS = 0.8	5.1,RP2D group 13.6	33	Completed	NCT03661515	-
Abboud et al.	Phase Ⅰ	40	R/R AML	X (60 mg, days 1, 5, 10, 12) combined with CLAG	60 mg BIW, days 1, 5, 10, and 12	18/40 (45%)	6.1	7.8	60.0	Completed	NCT02249091	—
Wang et al.	Phase Ⅰ	20	Newly diagnosed or R/R AML	X (60 mg and 80 mg BIW) combined with MA	80mgBIW, 2 weeks on, 2 weeks off	13/20 (65%), R/R AML 38%, newly diagnosed AML 83%	1-year PFS rate 68%	1-year OS rate 69%	57	Completed	NCT02573363	It is most promising when combined with cytarabine and anthracyclines
Bhatnagar et al.	Phase Ⅰ	23	R/R AML	X (30–55 mg/m^2^, days 1, 3, 8, 10, 15, 17) combined with MEC	30 mg/m^2BIW, days 1, 3, 8, 10, and 15	8/23 (35%)	—	8.5	30.4	Completed	NCT02299518	-
Sweet et al.	Phase Ⅰ	21	Newly diagnosed high-risk AML	X (60–80 mg BIW) combined with DNR (60 mg/m^2^, days 1–3) and Ara-C (100 mg/m^2^ days 1–7)	80mgBIW,3 weeks on, 1 week off	10/19 (53%)	—	10.3	31.6	Completed	NCT02403310	-
Timothy et al.	Phase Ⅱ	21 vs. 7	Newly diagnosed≥60 years AML	X (60–80 mg BIW) combined with DNR (60 mg/m^2^ days 1–3) and Ara-C (100 mg/m^2^, days 1–7) vs. DA regimen	60mgBIW,3 weeks on, 1 week off	18/21 (85%)vs. 3/7 (43%)	18.6 vs. 3.6	28.0 vs. 8.8	—	Completed	—	—
Janssen et al.	Phase Ⅱ	51 vs. 51	Newly diagnosed≥60 years AML	X combined with DNR (60 mg/m^2^, days 1–3) and Ara-C (200 mg/m^2^, days 1–7) vs. DA regimen	60mgBIW,4 weeks continuous	59% vs. 80%	18-month EFS rate 26% vs. 45%	18-month OS rate 33% vs. 58%	—	Completed	NL5748 (NTR5902)	—
Bhatnagar et al.	Phase Ⅰ	25	Newly diagnosed elderly or R/R AML	Decitabine followed by X (23–55 mg/m^2^, BIW)	0mgBIW,2 weeks on, 2 weeks off	8/25 (32%), R/R AML 20%, newly diagnosed AML 80%	5.9	5.9	—	Completed	NCT02093403	-
Daver et al.	Phase Ⅰ	14	R/R FLT3-mutated AML	X combined with Sorafenib	60mgBIW, 4 weeks continuous	4/11 (28.6%)	—	3.5	—	Completed	—	—
—	Phase I/II	—	Newly diagnosed AML	X + VA	60mgQW for 3 weeks, then 1 week off	8/8 (100%)	—	—	—	Recruiting	NCT05736965	Ongoing
—	Phase I/II	—	AML	X + VA	60mgQW for 2 weeks	—	—	—	—	Completed	NCT05736978	
—	Phase I	—	—	X + HAAG ± HMA	60mgBIW for 2 weeks, then 2 weeks off	—	—	—	—	Recruiting	NCT05805072	
—	Phase I/II	—	—	X + HAD or X + CAG	60mgBIW for 2 weeks, then 2 weeks off	—	—	—	—	Recruiting	NCT05726110	
—	Phase II	145	Newly diagnosed intermediate - high - risk MDS	X + Venetoclax + Chemotherapy	—	—	—	—	—	Recruiting	NCT04898894	
—	Phase I/II	—	—	X + Venetoclax + Azacitidine	60mgQW、40mgBIW or 60mgBIW continuously for 4 weeks	—	—	—	—	Recruiting	NCT06449482	

Notes: RP2D: Recommended Phase II, dose; R/R: Relapsed/Refractory; MDS: myelodysplastic syndromes; IDA: idarubicin; Ara-C: cytarabine; MA: Mitoxantrone + Cytarabine; FLAG: Fludarabine + Cytarabine + G-CSF; CLAG: Clofarabine + Cytarabine + G-CSF; DNR: daunorubicin; MEC: Mitoxantrone + Etoposide + Cytarabine; MRD: minimal residual disease; VA: venetoclax combined with azacitidine; BIW: twice a week; QW: once a week; CR: complete remission; CRi: Complete Remission with Incomplete Hematologic Recovery; PFS: Progression-Free Survival; EFS: Event-Free Survival; OS: overall survival; -: No data available.

Wang et al. (2018) treated 20 newly diagnosed or R/R AML patients with selinexor, high - dose cytarabine and mitoxantrone. No dose - limiting toxicities occurred and the overall response rate was 70%. Also, post - treatment p53 expression in bone marrow blasts increased, while FLT3 and Kit protein levels decreased, indicating a possible post - translational effect. In an American Association for Cancer Research Phase I trial, 21 newly diagnosed high-risk AML patients received selinexor with daunorubicin and cytarabine (7 + 3 regimen). The combination showed promise with a 53% CR/CRi rate and no dose-limiting toxicities during induction, suggesting benefit for high-risk patients and indicating that 80 mg of selinexor with the 7 + 3 regimen is safe (Sweet et al., 2020). For elderly newly diagnosed AML patients, two Phase II randomized controlled studies reported different results. Timothy et al. (Pardee et al., 2020) included patients aged 60 or older. Despite a higher ASXL1 mutation rate in the selinexor group (24% vs. 0), it had higher CR/CRi (85% vs. 43%) and a trend of longer median PFS. However, Janssen et al. (Janssen et al., 2022) studied patients aged 66 or older and found the selinexor-DA regimen led to higher grade 3+ infection rates and early mortality. Most patients reduced/stopped treatment early, with a CR/CRi rate lower than expected. Thus, for elderly or less tolerable AML patients, selinexor dosing needs cautious exploration to balance tolerability and efficacy.

AML patients with abnormal cytoplasmic Topo IIα localization can develop chemoresistance as chemotherapy drugs can’t effectively bind to form DNA cleavage complexes (Gravina et al., 2014; Turner et al., 2009; Turner et al., 2013; Mirski and Cole, 1995; Mutka et al., 2009). To address this, combinations of selinexor and topoisomerase II inhibitors (like etoposide, daunorubicin, mitoxantrone) are being studied. Preclinical trials showed significant improvement in leukemia mouse survival and leukemia burden reduction, indicating therapeutic synergy. Subsequent clinical trials using the MEC regimen (selinexor with etoposide, cytarabine, and mitoxantrone) tested 23 patients, achieving a 33% overall response rate and a median OS of 8.5 months (Bhatnagar et al., 2023), proving its therapeutic potential.

**FIGURE 3:**
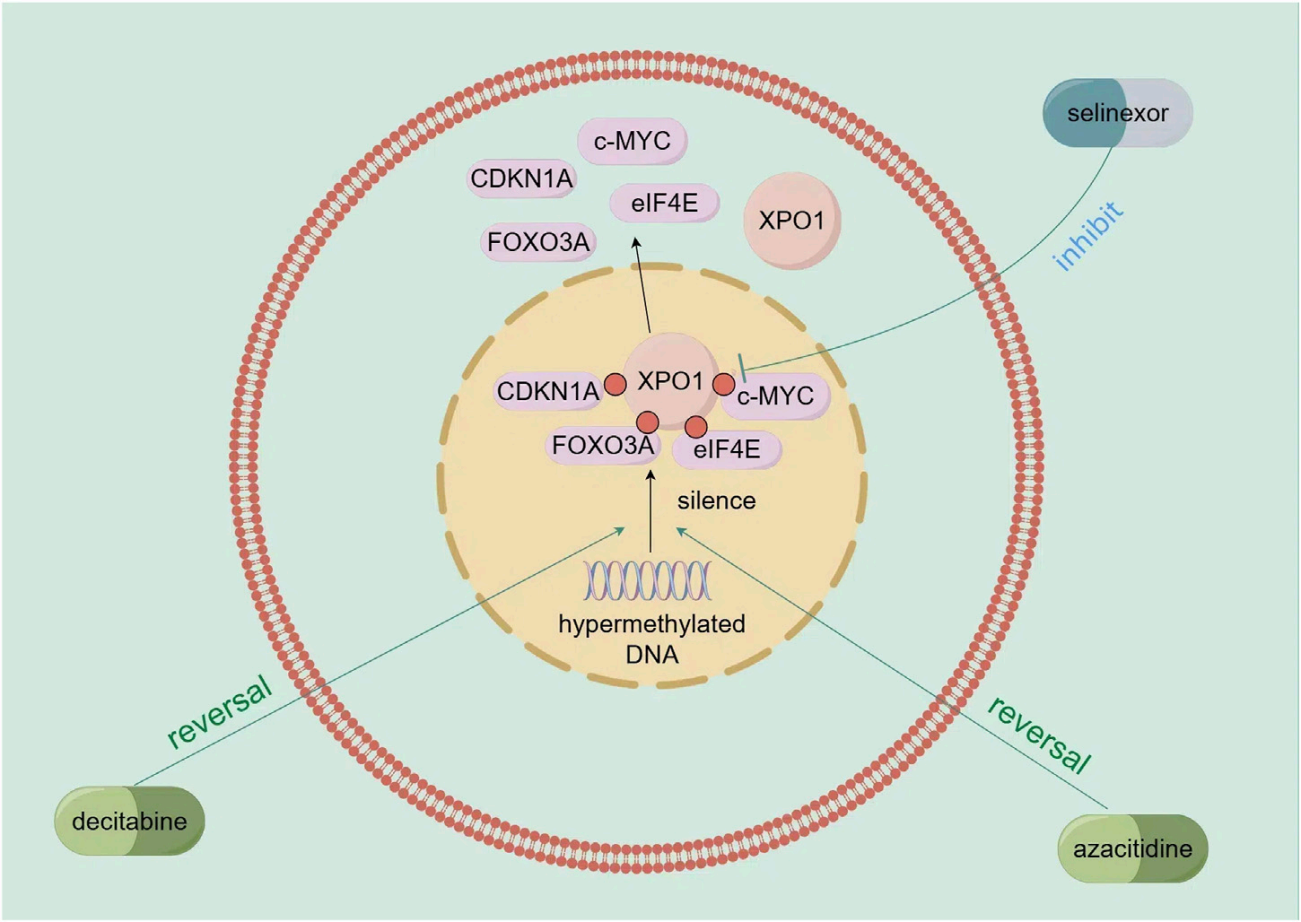
Mechanism of action of demethylating agents (decitabine and azacitidine) in combination with selinexor.

Researchers have explored combining selinexor with hypomethylating agents like decitabine and azacitidine. Decitabine reverses gene silencing from excessive DNA methylation, such as for CDKN1A and FOXO3A (Thépot et al., 2011; Schmelz et al., 2004), and these re - expressed tumor suppressor factors are regulated by XPO1 - mediated nuclear export (Mutka et al., 2009; Turner and Sullivan, 2008). Selinexor, an XPO1 inhibitor, blocks this export, accumulating the factors in the nucleus (Ranganathan et al., 2015). Azacitidine reverses abnormal gene methylation and promotes tumor suppressor gene re - expression (Fakih et al., 2018). Selinexor inhibits XPO1, blocking the nuclear export of tumor suppressor factors and cell cycle regulatory proteins like c - MYC (Fukuda et al., 1997). Together, they significantly downregulate XPO1, eIF4E, and c - MYC, inhibiting AML cell growth and promoting apoptosis (Long et al., 2023) (Figure 3). Preclinical trials showed that in the MV4 - 11 xenograft model, decitabine followed by selinexor increased mouse survival compared to selinexor alone (Ranganathan et al., 2015). In primary AML cells, decitabine pretreatment then selinexor reduced cell viability (Ranganathan et al., 2015). The selinexor - azacitidine combination synergistically reduced AML cell proliferation and promoted apoptosis by upregulating BAX and downregulating BCL - 2 (Long et al., 2023). These findings have been applied in clinical trials. A Phase I trial of decitabine plus selinexor for AML included 25 patients, with an overall response rate of 40%. Using decitabine followed by 60 mg selinexor twice weekly for 2 weeks improved tolerability, showing potential for high - risk AML (Bhatnagar et al., 2019).

**FIGURE 4:**
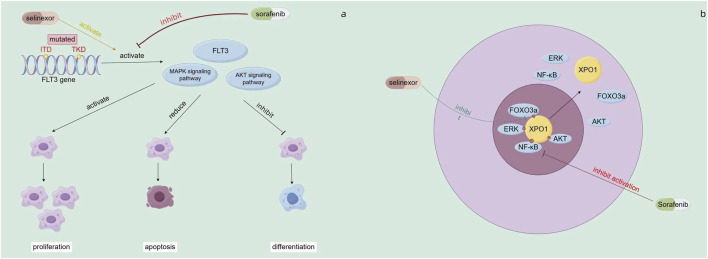
**(a)** Mechanisms of action of sorafenib **(b)** Mechanism of combined action of sorafenib and selinexor.

There has been progress in combining selinexor with FLT3 inhibitors. Selinexor, a selective nuclear export protein inhibitor (SINE), inhibits XPO1 (CRM1) to block the nuclear export of tumor suppressor proteins like p53, p21, and p27. In FLT3 - mutated AML cells (with ITD or/and TKD mutations), these mutations activate FLT3 and downstream MAPK/AKT signaling, promoting leukemia cell proliferation, inhibiting differentiation, and reducing apoptosis (Zhang et al., 2008) (Figure 4a). Sorafenib, a FLT3 - targeted drug, can inhibit this activation. The combination of selinexor and sorafenib increases the nuclear accumulation of ERK, AKT, NFκB, and FOXO3a, promoting apoptosis and differentiation (Zhang et al., 2018) (Figure 4b). *In vitro*, this combination promotes differentiation of FLT3 - mutated AML cells into early myeloid cells at low doses with minimal cytotoxicity. Under hypoxia, it partially reverses the protective effect of the hypoxic microenvironment on AML cells and induces synergistic apoptosis. In mouse models, the combination therapy improves mouse survival and reduces leukemia burden (Zhang et al., 2018). A subsequent Phase I study combined FLT3 inhibitors with sorafenib for R/R FLT3 - mutated AML. Among 17 patients, 29% achieved composite complete remission, with a median OS of 4 months. This shows good safety and clinical activity. Among AML patients with FLT3-ITD and/or D835 mutations, 5 (45%) achieved composite complete remission (CRc), and 2 of these patients had a negative FLT3-response at the time of therapy. (Daver et al., 2018).

Based on the published data to date, the dosage (dosage range of 100 mg once a week, 60–80 mg twice a week) and frequency (3 weeks on, 1 week off or 2 weeks on, 2 weeks off) of selinexor vary for AML patients with different tolerance levels and when combined with different intensity drug regimens. The total dose per course is 240–320 mg, and it is necessary to discontinue medication for at least 1 week to ensure recovery from myelosuppression. In addition to the above data, there are several ongoing clinical trials, as detailed in Table 2.

## 4 Limitations of selinexor

Although selinexor has shown good therapeutic effects in the treatment of AML, there are still some obvious adverse reactions that affect the quality of life of patients, and some patients are unresponsive to selinexor treatment or relapse after treatment. For the above situations, it is necessary to take control measures to improve the patient’s condition in a timely manner, and it is also necessary to conduct in-depth research and analysis on the resistance mechanism of selinexor.

### 4.1 Adverse reactions of selinexor

Data from the BOSTON study and the MARCH study show that selinexor has no cumulative toxicity and no organ toxicity, and the current FDA label does not have a black box warning (Grosicki et al., 2020; Qiu et al., 2022). In the monotherapy of AML with selinexor, it has been found that patients will experience certain adverse reactions when treated with selinexor, which can be divided into five categories: hematological, digestive system, systemic, biochemical, and other, with representative symptoms being:

(1) Digestive system adverse reactions: nausea, vomiting, and diarrhea (Garzon et al., 2017).
(2) Hematological adverse reactions: thrombocytopenia and neutropenia (Garzon et al., 2017).
(3) Systemic adverse reactions: fatigue and loss of appetite (Garzon et al., 2017).
(4) Biochemical adverse reactions: hyponatremia (Garzon et al., 2017).
(5) Other adverse reactions: weight loss (Garzon et al., 2017).

The above symptoms are the most common in adverse reactions, among which the most frequent is fatigue (60%), and the most frequent grade 3/4 adverse reaction is hematological (32%), so it is necessary to pay high attention to it in treatment and take corresponding measures in a timely manner (Garzon et al., 2017). Fatigue can significantly reduce patients’ quality of life, affecting their ability to perform daily activities and their adherence to treatment, which is more pronounced in elderly AML patients and may further exacerbate their frailty. Hyponatremia may impact patients’ neurological and cardiac functions, increasing the risk of falls, altered mental status, and arrhythmias. In elderly patients, it may lead to more severe consequences, such as cognitive decline and complications related to electrolyte imbalances. Hematologic toxicity, including thrombocytopenia and neutropenia, can lead to an increased risk of bleeding and infections. For elderly AML patients, whose bone marrow function is often already compromised, they may face more severe hematologic toxicity, such as prolonged cytopenias and difficulty in recovery, which may increase treatment-related mortality.

In the past treatment of multiple myeloma (MM) with selinexor, the categories and incidence rates of adverse reactions are similar to those in the treatment of AML, and control measures can be used to intervene in adverse reactions to ensure the prognosis of patients (Gavriatopoulou et al., 2020). Specifically, see Table 3. In terms of control measures, different drugs are mainly used for different symptoms, and supportive nursing measures, such as nutritional support and dose adjustment, can also be taken. The first occurrence of adverse events of selinexor is mainly concentrated within the first two treatment cycles (Nooka et al., 2022), and it is necessary to closely monitor the patient’s blood routine, blood biochemistry, weight, etc., in order to detect and actively handle them in a timely manner. Before medication, it is also necessary to educate patients to recognize the symptoms of selinexor-related adverse reactions and inform patients of the known toxicities, such as anorexia, nausea, and fatigue, which can help patients be prepared to deal with any imminent adverse events.

**TABLE 3:** Summary of common adverse reactions of selinexor (X) in the treatment of acute myeloid leukemia (AML) patients.

Type of adverse reaction	Specific symptoms	Overall incidence	Grade 3/4 incidence	Control measures
Hematological	Thrombocytopenia	20%	19%	Platelet transfusions and Thrombopoietin Receptor Agonists (TPO-RAs): Romiplostim or Eltrombopag
	Neutropenia	14%	13%	Granulocyte Colony-Stimulating Factor (G-CSF): Filgrastim or Pegfilgrastim
Gastrointestinal	Nausea	53%	—	5-HT3 antagonists, Neurokinin 1 receptor antagonists, Benzodiazepines, and Cannabinoid receptor antagonists, etc
	Vomiting	38%	5%	
	Diarrhea	39%	3%	Loperamide and Bismuth Subsalicylate
Systemic	Fatigue	60%	14%	Methylphenidate, etc
	Decreased appetite	53%	—	Megestrol, Cannabinoid drugs, or Olanzapine
Biochemical	Hyponatremia	22%	—	Sodium replacement therapy: Sodium tablets
Other	Weight loss	25%	—	Nutritional counseling: Providing nutritional support and dietary advice. Appetite stimulants: such as Megestrol, Cannabinoid drugs, or Olanzapine

Considering the mechanism of action of selinexor, its inhibition of XPO1 affects the nucleocytoplasmic transport of various signaling pathways and proteins within the cell, which may partly explain the occurrence of the aforementioned adverse reactions. For instance, inhibition of XPO1 may lead to changes in the intracellular distribution of certain proteins related to cell metabolism, immune regulation, and maintenance of the hematopoietic system, thereby causing issues such as fatigue and hematologic toxicity. In elderly AML patients, due to their declining physiological functions and reduced compensatory capacity, these adverse reactions may be more pronounced and more likely to lead to severe clinical consequences. Therefore, during the treatment process, the patient’s age and physical condition should be fully taken into account to develop personalized treatment plans. Close monitoring of adverse reactions is also essential so that effective intervention measures can be taken in a timely manner to alleviate patient discomfort and improve the safety and tolerability of the treatment.

In addition, other adverse reactions have been found when selinexor is used to treat other diseases, such as constipation, dyspnea, upper respiratory tract infection, etc. (Syed, 2019). Even serious adverse reactions have occurred, such as pneumonia (Gasparetto et al., 2019), lymphocytopenia (Chari et al., 2018; Chen et al., 2018), hyperglycemia (Gasparetto et al., 2019; Chen et al., 2018; Taylor et al., 2018; Gounder et al., 2018), hypocalcemia (Taylor et al., 2018), pulmonary infection (Taylor et al., 2018), hypotension (Taylor et al., 2018), etc., although the incidence rate is low, it still needs attention. The above symptoms still need to be closely monitored and detected in subsequent studies.

### 4.2 Resistance to selinexor

In experiments probing the resistance mechanism of selinexor, human fibrosarcoma HT1080 cell lines were subjected to gradually increasing concentrations of KPT-185 (an early analog of selinexor) for up to 10 months. This process yielded SINE-resistant cells. These resistant cells showed more than 100 times lower sensitivity to SINE compounds compared to the parent cells (Crochiere et al., 2015). Compared with the parent cells, the nuclear accumulation of tumor suppressor proteins in the resistant cells was reduced, showing an extended cell cycle (Crochiere et al., 2015); the changes in protein expression were similar, but the changes in the parent cells were more significant (Crochiere et al., 2015); although the drug treatment patterns of transcription changes in the parent and resistant cells were similar, the response in the parent cells was stronger (Crochiere et al., 2015).

Microarray analysis shows that in drug - resistant cells, several key signaling pathways change, including gene expression changes related to cell adhesion, apoptosis, and inflammation (Crochiere et al., 2015). These changes indicate that SINE resistance may be achieved by altering the signaling pathways downstream of XPO1 that are inhibited, among which the NF - κB and AKT - FOXO3 signaling pathways are particularly important.

**FIGURE 5:**
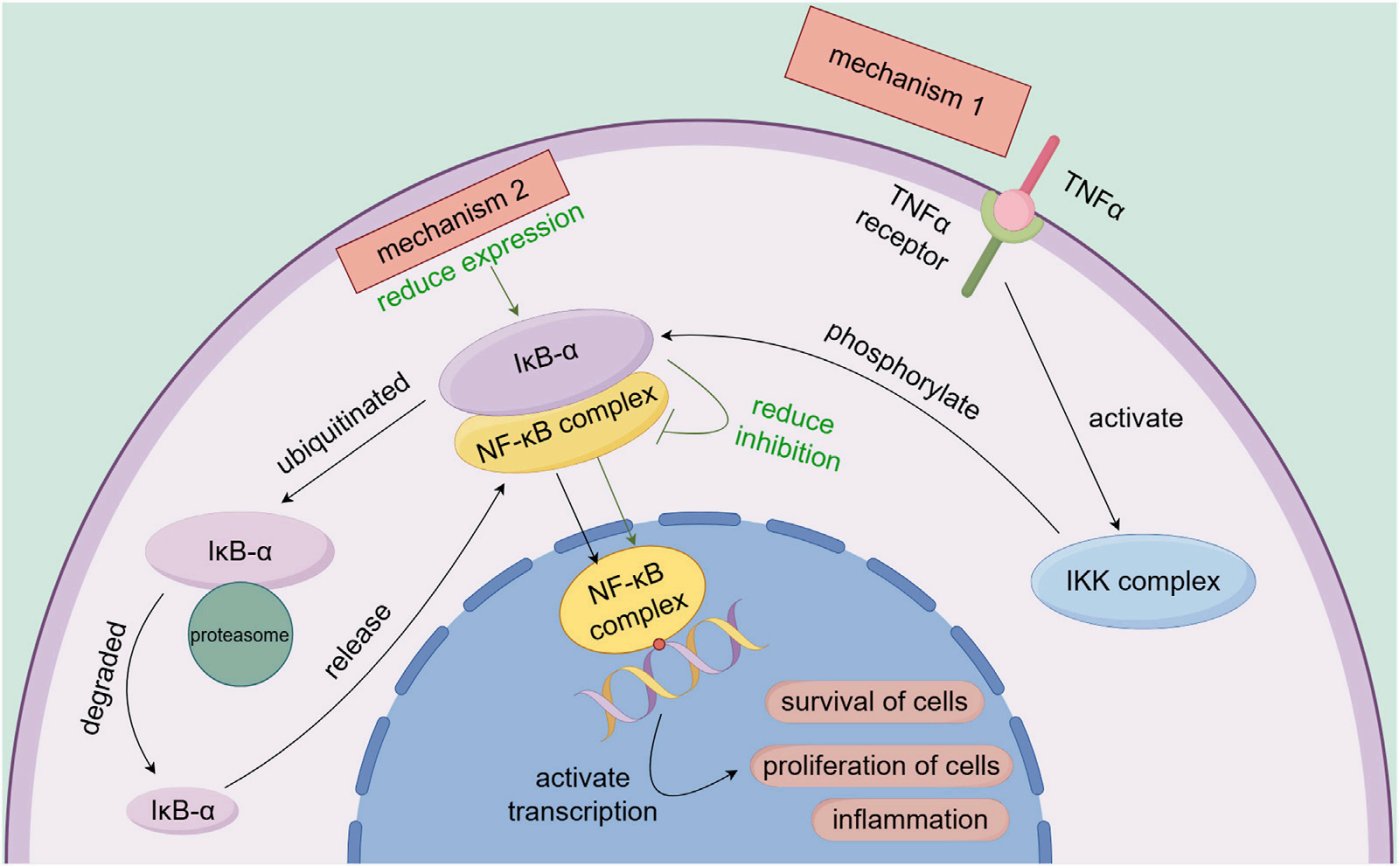
Association of the NF-κB signaling pathway with the mechanisms of selinexor resistance.

NF-κB is a key transcription factor regulating cell survival, proliferation, and inflammation. Normally, NF-κB binds to IκB-α and remains inactive in the cytoplasm (Traenckner et al., 1995). When stimulated (e.g., by TNF-α), IκB-α is phosphorylated and degraded, releasing NF-κB to enter the nucleus and activate gene transcription, promoting cell survival and proliferation (Johnson et al., 1999). Selinexor inhibits XPO1, blocking the nuclear export of IκB-α and the NF-κB p65 subunit. This causes their nuclear accumulation and binding, suppressing NF-κB activity, reducing NF-κB-dependent gene transcription, and exerting anti-tumor effects (Kashyap et al., 2016) (Figure 5).

**FIGURE 6:**
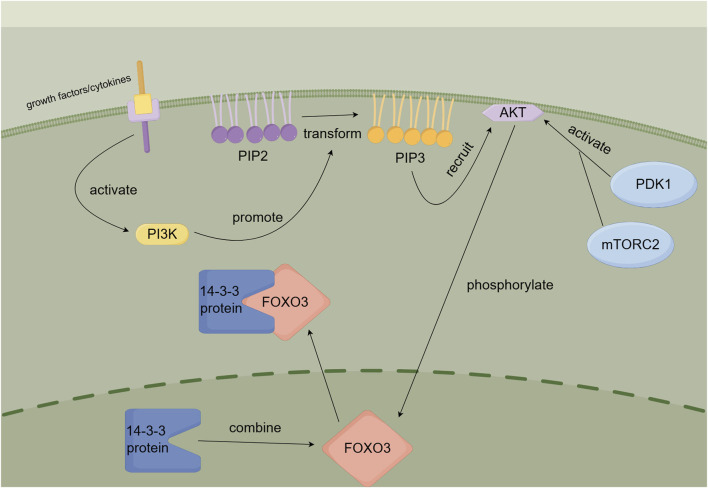
AKT-FOXO3 signaling pathway.

During selinexor therapy, AML cells may develop drug resistance by activating the AKT-FOXO3 signaling pathway. When AKT is activated, it phosphorylates FOXO3, particularly at the S253 site. This phosphorylation causes FOXO3 to bind to 14-3-3 proteins and remain in the cytoplasm, preventing its nuclear translocation (Brunet et al., 1999). As a result, selinexor cannot block the nuclear export of FOXO3 to induce pro-apoptotic transcription, significantly reducing its therapeutic efficacy (Emdal et al., 2022). Figure 6 illustrates this mechanism.

Moreover, Studies indicate that cancer cells may develop resistance to selinexor by upregulating alternative nuclear export pathways. For instance, overexpressing XPOT, a tRNA export protein, can facilitate tRNA nuclear export, bypassing XPO1 blockage. Similarly, upregulating KPNB1, a nuclear transport protein, enables essential nuclear-cytoplasmic molecular transport even in the presence of selinexor. This overexpression helps cancer cells escape selinexor’s inhibitory effects (Cohen et al., 2021). Research on selinexor resistance in CML treatment reveals gene expression changes in resistant cell populations, with distinct RNA expression patterns between resistant and parental cells. These alterations may impair selinexor’s ability to disrupt nuclear-cytoplasmic transport signals, reducing its efficacy. Resistant cells might also upregulate heat shock proteins to counteract selinexor-induced stress and protect cells (Cui et al., 2024). Additionally, a decrease in ferroptosis-related molecule expression may render resistant cells less sensitive to ferroptosis, while increased expression of autophagy-related genes may aid cell survival under selinexor treatment by effectively responding to stress and removing damaged components (Cui et al., 2024). Cell populations with stem cell traits may maintain stemness by upregulating specific genes, potentially contributing to resistance formation (Cui et al., 2024).

## 5 Comparison of selinexor with second-generation SINEs

In the field of AML treatment, selinexor has shown unique advantages and provided new therapeutic options for patients. Among the new generation of SINEs, KPT-8602 has also emerged as a promising candidate. Compared with selinexor, KPT-8602 has lower blood-brain barrier permeability, which reduces central nervous system-related adverse effects such as anorexia and weight loss (Etchin et al., 2016). KPT-8602 can be administered daily, while selinexor is limited to two or three times per week due to side effect concerns (Etchin et al., 2016). Additionally, KPT-8602 has demonstrated stronger anti-leukemia activity and less toxicity to normal hematopoietic stem cells and progenitor cells in both *in vitro* AML cell lines and *in vivo* patient-derived xenograft (PDX) models (Etchin et al., 2016). Currently, KPT-8602 has entered clinical trials for relapsed/refractory multiple myeloma (NCT02649790), and studies (NCT05918055) are ongoing to assess its combination with drugs such as Inqovi ((Decitabine-Cedazuridine)).

## 6 Outlook

Selinexor is showing great potential in the treatment of AML, especially for relapsed/refractory patients and the elderly. Its unique mechanism of action offers a new therapeutic approach, which is expected to enrich treatment strategies and promote the development of personalized medicine. However, to fully utilize its benefits, future clinical actions and research should focus on several key areas.

Optimizing selinexor’s treatment plan is crucial. Monotherapy is well - tolerated in elderly patients or those unable to endure intensive chemotherapy, with lower non - hematological toxicity. Some patients can achieve durable remission. Combination therapies can significantly enhance efficacy. For example, combining selinexor with chemotherapy (like cytarabine, daunorubicin) or targeted drugs (like FLT3 inhibitors, hypomethylating agents) can improve CR/CRi rates and prolong survival. The most remarkable effects are observed when combined with low - dose cytarabine and daunorubicin. Additionally, investigating other combination therapies that can synergize with selinexor is necessary. Emerging preclinical trials indicate that combining it with BCL - 2 (Pan et al., 2013), AKT (Lin et al., 2022), or EZH2 inhibitors (Al-Ghabkari and Narendran, 2021) (such as GSK126 (Huang et al., 2019), UNC 1999 (Konze et al., 2013), and EPZ - 5687 (Lindsay et al., 2017) shows potential to enhance selinexor’s efficacy and overcome monotherapy limitations.

The therapeutic strategies of selinexor are expected to be further expanded. For instance, the integration of RNA nanotechnology is anticipated to enhance selinexor’s targeting ability, reduce its impact on normal cells, and consequently improve therapeutic outcomes while minimizing adverse reactions (Lv et al., 2023). Additionally, drawing on the experience of using nanoparticles in lung cancer therapy, the development of selinexor-based nanoparticle formulations may improve the drug’s tissue targeting, reduce systemic toxicity, and offer AML patients a safer and more effective treatment option (Liu et al., 2023a). Meanwhile, the role of long-chain non-coding RNA (LncRNAs) in regulating myeloid-derived suppressor cells suggests that modulating LncRNAs to improve the immune microenvironment in AML and combining this approach with selinexor therapy could enhance treatment efficacy and create more opportunities for patients with relapsed/refractory AML (Liu et al., 2023b).

Managing selinexor - related adverse events is vital for improving treatment adherence and quality of life, especially in elderly patients. Fatigue is the most common adverse reaction (Gavriatopoulou et al., 2020). Patients can prevent it by balancing rest and activities, maintaining good nutrition, and managing stress (Gavriatopoulou et al., 2020). Other adverse reactions should be treated symptomatically. Regular monitoring of patients’ conditions and educating them about adverse reaction symptoms are also necessary.

Overcoming drug resistance remains a significant challenge. Research is needed to understand selinexor resistance mechanisms. Advanced technologies like high - throughput sequencing and transcriptomic analysis can identify resistance biomarkers, aiding in the development of new combination therapies and guiding patient selection for selinexor. Expanding clinical trials to verify selinexor’s efficacy and safety in diverse patient groups, including the elderly and those with specific gene mutations, can better define the target population and ensure its effective clinical use.

Finally, incorporating selinexor into clinical practice guidelines based on emerging evidence and providing education and training for healthcare professionals will help ensure its proper use and optimize patient management. By addressing these priorities, we can enhance selinexor’s clinical value, improve patient outcomes, and move closer to overcoming the challenges of AML.
